# Qualitative study to inform the design and contents of a patient-reported symptom-based risk stratification system for patients referred from primary care on a suspected head and neck cancer diagnostic pathway

**DOI:** 10.1136/bmjopen-2024-094197

**Published:** 2025-04-03

**Authors:** Abigail Albutt, Lynn McVey, Rebecca Randell, John C Hardman, Ian Kellar, Chinasa Odo, Jo Patterson, Paula Theresa Bradley, Claire Davies, Theofano Tikka, Vinidh Paleri, Nikki Rousseau

**Affiliations:** 1 Leeds Institute of Clinical Trials Research, University of Leeds, Leeds, UK; 2 University of Bradford, Bradford, UK; 3 Faculty of Health Studies, University of Bradford, Bradford, UK; 4 Royal Marsden Hospital NHS Trust, London, UK; 5 Department of Psychology, University of Sheffield, Sheffield, UK; 6 School of Health Sciences, University of Liverpool Faculty of Health and Life Sciences, Liverpool, UK; 7 Newcastle University, Newcastle upon Tyne, UK; 8 St George's University Hospitals NHS Foundation Trust, London, UK; 9 Surgical, Diagnostic and Devices Division, University of Leeds, Leeds, UK

**Keywords:** Patients, Head & neck tumours, Triage, QUALITATIVE RESEARCH, Digital Technology

## Abstract

**Abstract:**

**Objectives:**

This study aims to inform the development of a patient-reported symptom questionnaire for head and neck cancer and outline the requirements for a patient-reported symptom-based risk stratification system. The study objectives are to explore how clinicians ask questions and decide subsequent steps for patients referred with suspected head and neck cancer; the language patients and clinicians use to describe symptoms; how clinicians reassure and discharge low-risk patients; and identify clinician and patient experiences of the head and neck cancer diagnostic pathway and their views on a novel diagnostic pathway using patient-reported symptom-based risk stratification.

**Design:**

The study employed qualitative methods including observation and recordings of clinic consultations and semistructured interviews with clinicians and patients. Analysis proceeded concurrently with data collection using a rapid qualitative analysis approach.

**Setting:**

Three acute UK National Health Service Trusts with variation in service delivery models. Data collection took place between April and October 2023.

**Participants:**

One hundred and fifty-six adults referred for suspected head and neck cancer, and 21 clinicians from different subspecialties were recruited. A subset of recruited patients (n=16) and clinicians (n=13) were interviewed. One observation of a general head and neck clinic was conducted.

**Results:**

The findings highlight types of symptoms and the language used by patients and clinicians to describe these symptoms in clinic consultations. During interviews, patients described the need for in-person support and human clinical decision-making, an accessible system for reporting their symptoms and reassurance regarding the security of patient data. Clinicians discussed the need for risk scores to be sufficiently validated to be trusted, the potential clinical usefulness of a risk score-based system, for example, to support triage by discriminating symptoms, and accessibility for patients. The observation highlighted inconsistent and sometimes unclear referral information and the limited time clinicians have to read referral information.

**Conclusion:**

The findings have implications for the development of a patient-reported symptom-based risk stratification system. As well as ensuring patients can understand the language used, it will be important to consider how their emotional needs can be met. The findings also have wider implications for understanding the impact of language on emotionally evocative healthcare interactions.

**STRENGTHS AND LIMITATIONS OF THIS STUDY**

- This study forms part of a large multicentre study exploring whether the implementation of a patient-reported symptom-based risk stratification system for suspected head and neck cancer (sHNC) referrals improves patient safety and experience (the evolution of a patient-reported symptom-based risk stratification system to redesign the sHNC referral pathway programme (EVEREST-HN)).
- A key strength of the study is the use of multiple methods (observation, consultation recordings, interviews) to inform the development of a patient-reported symptom-based risk stratification system.
- An in-depth exploration from the perspective of multiple key stakeholders was achieved.
- Recruitment information was available in different formats and languages to ensure inclusivity.
- Only a small number of geographical locations were included in the study.

## Introduction

 Head and neck cancer (HNC) is an umbrella term for cancers of the nose, mouth, throat, voice box, thyroid and salivary glands. It is the eighth most common form of cancer in the UK and has increased in incidence by 20% in the last decade.^1^ Many patients present to their general practitioner (GP) and dentist with symptoms affecting the head and neck region, some of whom need specialist assessment in the hospital via a dedicated suspected HNC (sHNC) referral pathway. In England, in 2022/2023, 275 354 patients were referred with sHNC, making it the fifth largest group of suspected cancer referrals.^1^

The symptoms associated with sHNC (eg, a feeling of a lump in the throat) are common and can be difficult to distinguish from other conditions. As a result, large numbers of patients with sHNC are referred from primary care for specialist opinion into secondary care services, which must be configured to meet this demand. Given the NHS cancer plan’s aim to diagnose cancer sooner, the number of sHNC referrals over the last 10 years has risen from 140 to 404 patients per 100 000 population.^2^ Standard practice in the UK is currently for all sHNC referrals to be offered a face-to-face consultation as their first hospital contact, in chronological order of referral, although only 3–5% of referrals are diagnosed with cancer. Unfortunately, partly due to capacity issues, 1-in-10 sHNC referrals are not seen within the 2-week target.^3^ Patients have reported significant anxiety during this period while waiting for their appointment, making any delays here very undesirable.^4^ Anxiety has also been reported among HNC patients throughout the whole diagnostic and treatment pathway.^4^,^6^

The sHNC pathway is different from other cancers, which usually apply to one organ (eg, prostate, breast) and may have screening tests available (eg, colon, breast) to screen referrals to improve efficiency.^7^ However, the diversity of subtypes of HNCs, combined with the fact that up to one-third of cancers diagnosed in this cohort are non-HNC but with head and neck symptoms,^8^ makes finding a single solution challenging.^9^

Our previous work has focused on the development of a Head and Neck Cancer Risk Calculator (HaNC-RC) symptom inventory, a risk assessment tool for use by specialists to aid referral of high-risk patients to urgent specialist clinics.^10^ HaNC-RC-v2 was deployed as a national service evaluation of sHNC referrals undergoing remote triage in secondary care during the initial peak of the COVID-19 pandemic and demonstrated early evidence of safety for this approach.^9^ This work demonstrated that clinicians could perform symptom-based remote triage, supported by risk stratification, and medium-term outcomes were acceptable. However, this algorithm was developed using symptom data recorded by clinicians after face-to-face consultation, where examination may have biased the symptom inventory reporting.^10^,^12^

The evolution of a patient-reported symptom-based risk stratification system to redesign the sHNC referral pathway (EVEREST-HN) programme proposes a novel approach to redesign the sHNC pathway that may enable earlier diagnosis of cancer or earlier reassurance that the patient is cancer-free. A central component of the pathway is the implementation of the SYmptom iNput Clinical (SYNC) system that contains both clinician-facing and patient-facing components and allows patients (with help from family/carers where needed) to complete an electronic questionnaire (the SYNC Symptom Questionnaire) at the time of referral, which will facilitate risk stratification before the hospital visit. Implementation of SYNC could permit higher-risk patients to be prioritised, target investigations to be arranged directly before patients are seen in clinic and potentially enable assessment for lower-risk patients via alternative pathways (eg, speech and language therapy-led clinics).

### Aims and objectives

This study aims to inform the development of a patient-reported symptom questionnaire for HNC and outline specification requirements for a SYNC system. The study objectives are

1. To explore:
  - How clinicians ask questions and decide subsequent steps for patients referred with sHNC;
  - The language patients and clinicians use to describe symptoms;
  - How clinicians reassure and discharge low-risk patients;
  - Clinician and patient experiences of the current diagnostic pathway and their views on the proposed SYNC system.
2. To use information from objectives 1 (a)–(d) to outline key requirements and appropriate language for use in a SYNC system and SYNC symptom questionnaire.

## Methods

### Study design

Multiple qualitative data collection methods were used to develop a comprehensive understanding of current practice within the sHNC pathway at the trusts (using interviews, observations and recordings of clinic consultations), the language used during consultations (recordings of clinic consultations) and the context within which care is delivered (interviews with key stakeholders). Rapid qualitative analysis of these data informed the requirements for a patient-reported symptom-based risk stratification system for sHNC referrals.

Participants were adult patients without a previous history of HNC attending a sHNC appointment at three NHS trusts that deliver a diagnostic service for the sHNC and clinicians of various grades conducting diagnostic consultations with these patients. Participants were willing and able to give informed consent for participation in the study. A subset of the patients and clinicians were purposively sampled from the wider sample to participate in a qualitative interview. Purposive sampling was based on demographic information including age, ethnicity, socioeconomic status and presenting symptoms and to ensure that each study site was represented.

Data collection took place between April and October 2023.

### Data collection

Data collection comprised non-participant observation and audio recordings of diagnostic consultations, patient interviews and clinician interviews.

Our methods are described in detail in our published protocol.^13^ In summary, members of the local clinical team proactively identified eligible patients through triage of referral letters. Eligible patients were those allocated to attend a diagnostic clinic or with symptom(s) or other characteristics identified as being important to include in the sample, for instance, a variation in age, ethnicity, socioeconomic status and presenting symptoms. Where possible, the patient information sheet was sent to patients with appointment details. Participants were approached and consented by local care teams, and written consent for consultations to be recorded was taken immediately prior to appointments. Verbal consent was taken and recorded prior to interviews conducted remotely. The recruitment strategy was co-developed with patient and public involvement (PPI) representatives and with reference to National Institute for Health Research INCLUDE guidance.^14^ Recruitment information was available in different formats and languages.

A subset of recruited patients and clinicians were interviewed by AA and LM, female, PhD, academic researchers with experience in applied health and qualitative research. Participants were all unknown to the researcher at the time of interview. Interviews were conducted remotely via Microsoft Teams using the telephone call function with patients and meeting function with clinicians. Separate semistructured interview schedules were developed for patient and clinician interviews with input from EVEREST-HN PPI representatives (see online supplemental appendix A for topic guides). Patients were invited to discuss their recent experience of the current diagnostic pathway and their views on the proposed SYNC system. Clinician interviews focused on the current diagnostic pathway, clinical decision-making processes prior to and during consultations and their views on the proposed SYNC system.

### Patient and public involvement

Two experts by experience, Chris Elkington and John Holmes, contributed to the conceptualisation of the study, protocol development and the development of the participant recruitment strategy and commented on patient-facing materials. Regular meetings (at least twice a year) were held throughout study conduct with CE, JH, our PPI lead (JP) and other authors as appropriate.

### Ethical considerations

A favourable ethical opinion for the study was given by North West - Liverpool Central Research Ethics Committee on 19 October 2022 (IRAS project ID: 314496).

### Data analysis

All interviews and consultations were audio-recorded, and interviews were transcribed verbatim. One consultation that was conducted in Urdu was translated. Transcripts and contemporaneous field notes from non-participant observation in clinical settings were edited to ensure participant anonymity. The analysis proceeded concurrently with data collection using an inductive rapid qualitative analysis approach and was conducted according to standard procedures of rigorous rapid qualitative analysis.^15 16^ Separate Rapid Assessment Procedure (RAP) sheets^15^ were developed by AA and LM for patient interviews, clinician interviews and consultations to record descriptive information from the transcripts and how it integrated into the developing analysis. The descriptive information recorded was based on the study objectives, for instance, consultation RAP sheets included headings to capture terms used by patients to describe symptoms and language used by clinicians that was helpful in generating relevant responses from patients (see online supplemental appendix B for templates of the interview and consultation RAP sheets). Researchers (AA and LM) completed RAP sheets after each interview and for each recorded consultation. Data relevant to all objectives were generated from all the data sources (interviews; consultations) although the focus varied across the different sources of data (eg, language used in consultations; views and experiences in interviews).

Clinic consultation RAP sheets were grouped by categories such as type of symptom, treating clinician and patient demographics. Summaries of the RAP sheets in these categories were developed (by AA) to identify patterns within and across categories. A purposive subset of consultations was then selected from the grouped categories for more detailed analysis, with the aim of including consultations relevant to all the HaNC-RC question areas, and variation in terms of patient demographics and consulting clinician. Content analysis of consultation recordings focused on language used, generating examples of terms used by patients to describe their symptoms, and aimed to identify language used by clinicians which is helpful in generating relevant responses from patients, as well as terms that seem to be poorly understood. Interview RAP sheets were analysed for similarities and differences, between and within the clinician and patient perspectives. Rapid qualitative analysis was used to develop requirements for a SYNC system that will be further developed in a subsequent work package. Site-level summaries were developed for each of the three sites to describe the process for organising clinics within the urgent sHNC pathway at that trust. We held ‘data clinics’ where the research team (AA, LM, NR, JP, VP, IK, CO, RR, JH, SC) exchanged interpretations of key issues emerging from the data. These facilitated decisions regarding data saturation.^17^ We considered data saturation to be reached when new perspectives or insights were no longer identified from interviewing further participants; further data were not generating new requirements; and the requirements identified were easily exemplified in the collected data. The study followed the Consolidated criteria for Reporting Qualitative research.^18^

## Results

One hundred and fifty-six adults referred via their GP or dentist for sHNC, and 21 clinicians of various grades and different subspecialties (ear, nose and throat (ENT) and oral maxillofacial) were recruited across three acute NHS trusts that deliver a diagnostic service for the sHNC referrals from primary care. Sites were recruited to include different service delivery models and unit size and to ensure a broad mixture of social, economic and cultural backgrounds of potential participants. The 156 clinic consultations recordings included a diverse group of patients in terms of ethnicity (although unfortunately we were unable to obtain these data at some sites) and included underrepresented groups such as people without permanent housing (see table 1 for patient demographics). The sample also represented the range of symptomology typically seen within a sHNC pathway. We had planned to conduct targeted recruitment of patients to ensure coverage of the full range of symptoms included in the HaNC-RC-v2. This proved difficult to achieve in practice, because of a combination of practical issues (adding complexity to recruitment for busy site staff) and variation in the quality and quantity of referral information.

**Table 1:** Characteristics of the patient sample

Characteristic	Percentage of sample
Sex	
Female	55.5%
Male	44.5%
Age	
18–30	5.8%
31–40	9.7%
41–50	17.4%
51–60	23.2%
61–70	21.9%
71–80	14.2%
81–90	7.7%
Ethnicity	
Not stated	49.7%
White	41.3%
Asian: Pakistani	2.6%
Asian: Bangladeshi	1.3%
Black: Caribbean	1.3%
Other Asian background	1.3%
Asian: Indian	0.6%
Black: African	0.6%
Mixed: White and Asian	0.6%
Other Black background	0.6%

A subset of 16 patients (gender, 56% women; age, 6% 18–30 years; 13% 31–40 years, 13% 41–50 years, 25% 51–60 years, 19% 61–70 years, 6% 71–80 years, 19% 81–90 years; ethnicity, 31% White, 69% not stated) were interviewed. All patients in the interview sample were referred to the urgent sHNC pathway via their GP (as opposed to dentist), but presenting symptoms included ENT and oral maxillofacial symptoms. Thirteen clinicians (three consultant head and neck surgeons, three consultant ENT surgeons, two consultant head and neck/thyroid surgeons, one ENT associate specialist, three ENT registrars, one consultant oral and maxillofacial surgeon) were interviewed. The average length of patient interviews was 25 min (range: 13 to 60 min), and clinician interviews were 30 min (range: 19 to 38 min). A non-participant observation of a general head and neck clinic at one site was conducted lasting 2 hours and 10 min. We did not conduct more observations for several practical reasons. We established that similar information could be captured via interviews with clinicians as opposed to having a de-brief in person after their consultations which proved to be infeasible due to time constraints. Also, as the study progressed, we saw that clinicians were able to audio-record their consultations without difficulty or the need for a researcher to be present. Finally, we felt that not having a researcher present during consultations was less intrusive for patients and less emotionally demanding for the researchers.

### Optimising a patient-reported symptom questionnaire for head and neck cancer (HNC)

The findings showed that clinicians ask patients questions during clinic consultations that align closely with items within HaNC-RC-v2. However, information about additional symptoms not included in HaNC-RC-v2 was sought during consultations, along with further probing and clarifying questions for all symptoms. Table 2 outlines the HaNC-RC-v2 items, how this information was sought by clinicians in a face-to-face consultation setting and examples of patients’ responses (see online supplemental appendix C (online supplemental table 1)) for further examples of language used by clinicians and patients). The language and terms used by clinicians and patients to describe symptoms that were identified from clinic consultation recordings were sense checked during interviews with a subset of clinicians.

**Table 2:** Suspected head and neck cancer symptomology and language used by patients and clinicians to describe symptoms

HaNC-RC-v2 items	Examples of questions asked by clinicians during consultations to understand patient lifestyle and elicit symptoms	Examples of terms used by patients to describe their lifestyle and symptoms during consultations
Unintentional weight loss (yes/no)	Have you lost any weight unintentionally since it’s [symptoms] come on? (B001)Have you lost any weight? Is your appetite good? (B002)	When asked if he’d lost weight intentionally, patient B023 and his girlfriend laugh, and he says ‘nooo, I’ve put it on!’Yes, I am losing a bit of weight which is a good thing as I have been overweight. My waist has gone from a 40 to a 38. (A040)
Smoking status (never smoked, ex-smoker, current smoker)	At the peak, how many a day did you smoke? (A009)‘Are you or were you ever a smoker?’ ‘No, not a smoker’. Later, when examining the mouth, sees staining and asks: ‘Have you ever chewed paan and do you still chew?’ Yes. (B001, patient B007)	Clinician (A037) asks, when did you stop smoking? Erm, 40 years ago.
Alcohol status (≤14 units/week, >14 units/week, ex-excess)	Clinician just asks ‘alcohol?’. Patient says only if she goes out, and clinician says ‘minimal’. (B013)Do you drink any alcohol? How many units would you say you drink in a week? (B009). Ever been a big drinker? (A005)	I don’t know units-wise (clinician asks ‘just in general?’). I’d say two bottles of wine and a couple of lagers [per week]. (A044)I drink a bottle of wine at the weekend, that’s it. (C017)
Hoarse voice (no, intermittent, persistent explained, persistent unexplained)	‘When did you first notice problem with your voice?’ ‘How long have you had this for?’ ‘Do you lose your voice completely?’ ‘So, once it comes, how long does it take to improve?’ (B013)Your voice sounds ok. Is that normal for you?’ (B001)	Patient describes voice as a bit ‘gravelly’. (B034)My voice feels hoarse if I’ve been talking much; you can hear it a bit now. (B022)My voice feels deeper and weak. (C053)
Sore throat (no, unilateral intermittent, bilateral/midline intermittent, unilateral persistent, bilateral/midline persistent)	Do you get a sore throat for a long period of time? (A037)Which side is the soreness? Did it start with a cold? When did you have the dental treatment? (A005)	A bit of sharpness but just on one side. (A045)It’s making my throat sore, very sore, and I’m often losing my voice. (B032)
Difficulty swallowing (dysphagia) (no, intermittent, persistent)	Do you think things sometimes go down the wrong way? (C013)Are you eating and drinking ok? As in, swallowing ok? (C006)Can you eat and drink whatever you want? (C013)	When I swallow…you know, like a muffin, I can’t even swallow that. It seems as if it gets stuck *there*. I always need to drink. (B035)When I’m swallowing, it almost gets a little bit tighter and it’s almost hard to clear my throat properly. I feel like I’ve got something stuck in there’. (A025)
New neck lump (no, fluctuating/reducing, persistent)	I know you’ve got a sensation of something, but have you ever actually felt a lump with your fingers? (B001)Have you had any glands come up that you can feel? (A005)	A few times I’ve felt a wave of pain inside [the lump]… something like a pinch [in the lump]. (A017)You get paranoid with every little lump and bump…it feels symmetrical but tender. (A045)
Pain on swallowing (odynophagia) (yes/no)	‘Is it painful when you swallow?’ ‘Would you describe it as discomfort?’ Clinician checks which side: ‘It’s only on the right? (B009)Do you have pain on swallowing? (A037)Is it tender when you swallow? (A005)	Patients generally give short answers either yes or no.
Oral ulcer/oral swelling (yes/no)	Are the ulcers painful? How long did pain last for? How long did ulcers last for? Did they reoccur in a different site? Does it bleed? Does it affect your speech? Anything that would have made the ulcers reoccur or worse, any food or anything you’re allergic to? (A019)	‘I had a hole at the side of my tonsillitis…I thought it was infected, I thought it was tonsillitis’. (A039)There’s a cut on my tongue, on the side of my tongue that’s been there for a while. (B032)
Unilateral ear pain with normal ear examination (yes/no)	‘Have you had any pain in the ears? Which side?’ ‘Is that on and off or constant?’ (B009)Are you getting any shooting pains in the ear when you swallow? (C013)	Patient describes ear pain as ‘jabbing’. (B035)My ears are blocked. (A034)My ears are itchy sometimes. (B041)
Noisy breathing (stridor) (yes/no)	No examples in consultations.	No examples in consultations.
Persistent head and neck skin lesion (yes/no)	No examples in consultations.	No examples in consultations.
Feeling of something/lump in throat (yes/no)	Is it [lump in throat] worsening or the same? (B002)Has it changed in a year? (C013)	When I’ve had my dinner, a couple of hours later, if I cough, there’s like bits of food still there. (C014)I could feel a lump on the inside of my throat. (B045)

HaNC-RCHead and Neck Cancer Risk Calculator

Lifestyle questions about weight loss, smoking and alcohol consumption were usually asked towards the start of the consultation to build a picture of these known risk factors for HNC and the patient’s general wellness. Clinicians asked detailed questions in the consultations regarding smoking and consumption of nicotine in other forms such as paan and betel nut. Clinicians asked about alcohol consumption in different ways. Most asked patients how many bottles or drinks they have a week rather than using units as a measure. There was sometimes a sense that the clinician found it awkward to ask patients about their alcohol consumption, and humour was used by clinicians and patients to navigate the topic, for example, a patient (A041) estimates how much she drinks: ‘about 4 Bacardi’s on a Friday and Saturday night’. The clinician (A037) misinterprets and says, ‘so 4 Bacardi’s a week’—the patient replies, ‘no on a Friday and Saturday so that would be 8…’. The patient jokes, ‘go on put 10 to be a devil’ (A041).

Information about some symptoms was easily sought and understood by clinicians during consultations, for example, patients seemed to understand and respond to questions about having a hoarse or ‘gravelly voice’. More clarification was needed when eliciting information from patients about most other symptoms. Clinicians used supplementary questions and often asked about the timeframe in which the patient has been experiencing the symptom, severity of pain caused by the symptom, and explored other possible causes for symptoms, for example, ‘have you had a cough or cold prior to your sore throat?’. Patients often gave information about multiple overlapping symptoms suggesting that they were not able to distinguish between some of their symptoms. For example, questions about having a feeling of something/lump in the throat often led patients to give information about other symptoms such as difficulty swallowing or a sore throat. This contributed to the need for clarification. There were a small number of consultations where a close family member spoke on behalf of the patient (in some cases without conferring with the patient). These patients tended to be elderly or not have English as a first language (one of these consultations was carried out entirely in Urdu and subsequently translated to English). There will likely be instances where the SYNC Symptom Questionnaire will be completed solely by a family member on behalf of the patient, rather than by the patient with support from a family member.

Some symptoms included in HaNC-RC-v2 were not present in consultations, for example, we found no examples of clinicians asking patients about stridor (noisy breathing) in our sample, suggesting it is not asked routinely as part of the consultation, although clinicians may hear stridor and not need to explicitly ask about it. Also, stridor symptoms can present as an emergency rather than via referral to a sHNC pathway. Conversely, there were symptoms explored in consultations that are not included in HaNC-RC-v2. For example, there were patients who experienced nasal problems and clinicians elicited information from patients about these symptoms.

Clinicians engaged in much emotional labour in consultations – reassuring, building rapport, empathising as well as giving information and clarifying. For example, one clinician (A019) asked what name he should use for the patient (A029). The patient replies, ‘full name, short version, whatever’s easiest for you’. Later, when taking the patient history, the clinician asks, in a gentle tone: ‘Do you work at all… (name)?’ He used the shortened version of the patient’s name here with a gentle tone of voice to convey he understands this might be a difficult issue for a young person with addiction issues.

### Patient and clinician interviews: requirements for a proposed SYmptom iNput Clinical (SYNC) system

#### Patient perspective

Some patients expressed concerns about whether people subject to digital exclusion due to a lack of access to technology and capacity to use it would be able to complete the SYNC Symptom Questionnaire. Interestingly, while some older patients felt confident in using technology, some younger patients felt it might exclude older people. Overall, those patients who did not feel confident using computers or smartphones ranged from middle to older age and talked about a reliance on others to help them if help is available. Having different methods available to complete the SYNC Symptom Questionnaire was suggested by several patients, for instance, answering the questions on the telephone with a health professional. Patients felt it would be important to give people information about the availability of different options to complete the questionnaire, such as via telephone call or text, at the start of the SYNC system so they can select their preference.

Patients had mixed feelings about whether completing the SYNC Symptom Questionnaire would create anxiety for themselves and others, while they wait for their urgent specialist appointment. Some patients had a non-anxious response and described themselves as pragmatic: ‘if a patient is seeking solutions to their symptoms, why would they be anxious answering questions?’ (A047). These responses tended to be in older people with comorbidities, males or those with a background working in healthcare. Others were more outwardly anxious, and some described themselves as ‘Googlers’ who might search the internet for information about the symptoms within the SYNC Symptom Questionnaire to determine how serious their own symptoms were, which could exacerbate their anxiety: ‘if I answered yes to a question, I’d be Googling what does this mean? I think it would just introduce a *lot* more anxiety’ (A028). Several patients highlighted the need for inclusion of links to sources of in-person support within the SYNC system to alleviate anxiety.

Patients stated that the SYNC Symptom Questionnaire must capture all patients’ symptoms, although acknowledged that this can be difficult to achieve. In one person’s experience, questionnaires tended to limit you to answer in a way that might not exactly reflect your symptoms, producing inaccurate information. Another patient felt straightforward questionnaires might not address everyone’s needs: ‘a questionnaire is not all encompassing and not everyone fits into that box’ (C043). The inclusion of a free text box to write your own account was suggested, although there is a limit to how much information you can give using a device or smartphone compared with in-person. Most patients wanted to have an in-person clinic appointment with a health professional after completing the SYNC Symptom Questionnaire. Patients appreciated the empathetic and reassuring approach clinicians had during consultations, and it helped reduce anxiety; they did not think this could be done by a computer when completing a symptom questionnaire.

**Figure 1:**
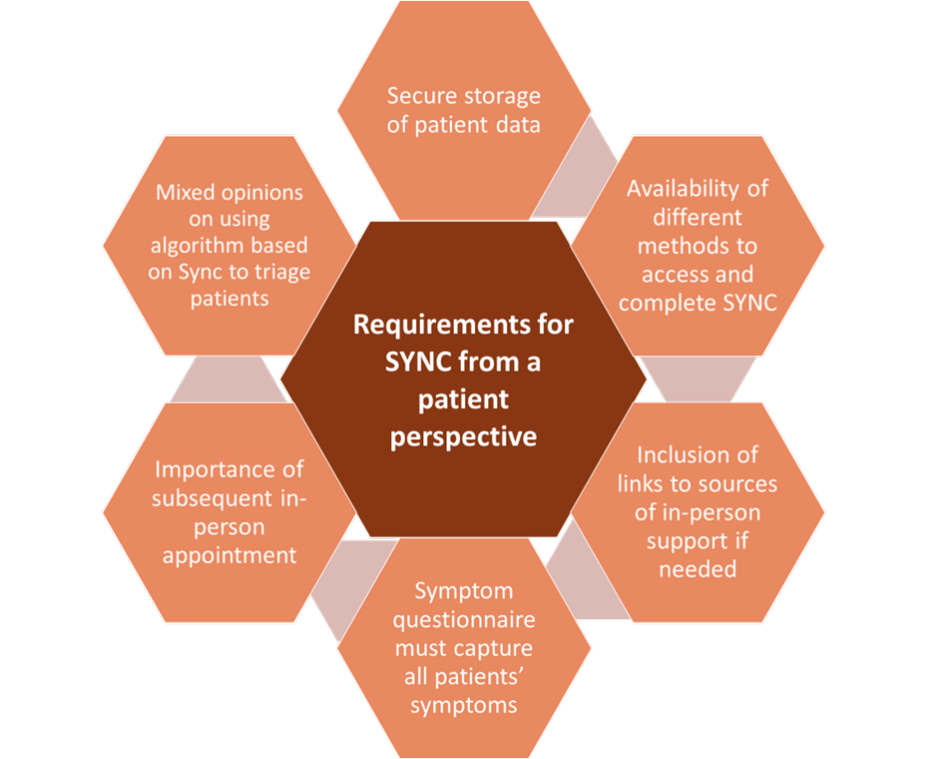
Requirements of a patient-reported symptom-based risk stratification system for suspected head and neck cancer (SYNC) from the patient perspective.

Most patients wanted reassurance that a clinician would review and act on computer-mediated decisions for triage and treatment decisions. They felt symptoms might be missed or misinterpreted: ‘there’s a risk for cases to be missed, if there were certain things that weren’t asked…I’m not sure how safe that would be’ (C001). Some felt that the use of computer-aided risk scores was impersonal and that might make people feel more anxious, particularly those with more concerning symptoms. Others felt that triage should not be based on the SYNC Symptom Questionnaire alone but that it could be useful to order tests for patients more quickly. Clinicians can consider ‘nuances’, questioning and confirming patients’ symptoms in a way that a computer cannot: ‘it’s the nuances that are important, and the interpretation of language and how the language is used, which can’t be done on a computer’ (B022). Patients highlighted the need for a clear understanding about who would see, and be acting on, their SYNC Symptom Questionnaire answers. This was important to provide patients with reassurance about the security of their data and to give them an understanding of their onward care (see figure 1 for requirements of a patient-reported symptom-based risk stratification system from the patient perspective and online supplemental appendix D (online supplemental table 2) for exemplar quotes for patient and clinician requirements).

#### Clinician perspective

**Figure 2:**
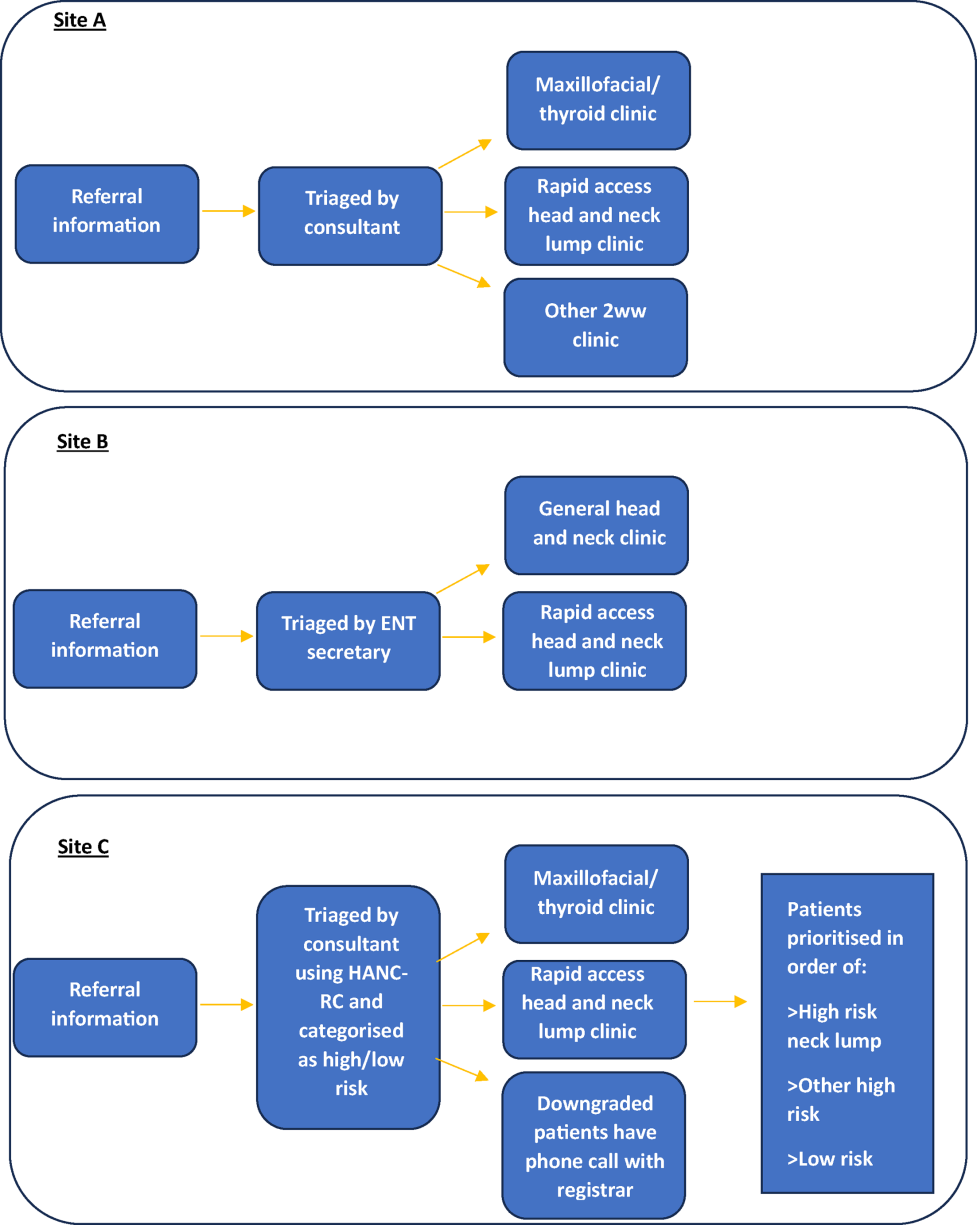
Suspected head and neck cancer (HNC) pathway at study sites—organisation of care. ENT, ear, nose and throat; HaNC-RC, Head and Neck Cancer Risk Calculator.

Clinicians could see a role for the SYNC system, although a few thought most patients were seen within the 2-week target which may obviate the need for the SYNC system. Most clinicians interviewed were aware of the HaNC-RC-v2 questionnaire and had either used it to triage patients during COVID-19^9^ or were using a version of it within their current triage processes (see figure 2 for urgent sHNC pathway at each site). Clinicians felt that it was important to know how low-risk patients were stratified using a risk score based on the SYNC Symptom Questionnaire. They did not think that a risk score would be used to determine whether a patient is seen in clinic, but that it could be useful to see the most vulnerable or at-risk patients more quickly. The patient-reported symptom information provided in the SYNC system could support the organisation of more targeted clinics: ‘I do think it will help us gain increased granularity in the subtypes of clinics that we offer, in order to make as many patient attendances one-stop as possible’ (B001).

One clinician talked about using the SYNC system to help effectively triage by discriminating symptoms: ‘if we can make our one-stop clinic fully one-stop and this triage can support that by the appropriate wording of questions, that would be important. And I think wording cannot be underestimated. And a good example of that in this process is how GPs will tick a neck lump box on a referral form very freely. But that form does not discriminate whether there is a visible or feelable, palpable neck lump’ (B001). Other clinicians echoed the view that a lack of detailed information received from primary care regarding patients’ symptoms made timely and efficient triage more difficult in secondary care, although there was acknowledgement that primary care clinicians are not experts and are referring in for an opinion.

Some concerns were highlighted about the possibility that patients may not be truthful in their answers to the SYNC Symptom Questionnaire to ensure they are given a face-to-face clinic appointment and seen sooner. It must be made clear to patients that the SYNC Symptom Questionnaire ‘isn’t being used to not get you seen…it’s been done to make sure you are seen by the correct person in the correct clinic’ (C006). One clinician thought patients’ answers to the SYNC questionnaire would be used as another stream of information alongside GP referral to triage unless there was sufficient validated evidence for it to be used as a triaging tool.

Clinicians noted that there needs to be the capacity to request tests prior to consultations and ensure patients are suitable for tests (eg, that they are not taking any anticoagulants) if tests are to be based on the SYNC Symptom Questionnaire. Some clinicians did not think this was practical. One clinician noted that some patients need to be examined before appropriate tests can be requested. There is a 9-day turnaround for scans at their site: ‘so, to make that more stretched when you might have avoided a scan if you saw them face-to-face, that seems counterintuitive’ (A008). Clinicians reported that they had a very limited amount of time to read referral information before they see a patient for a clinic consultation. They felt that the SYNC system must be automated before the patient comes into the clinic. Some clinicians felt that it was also important to receive the information the patient gives in the SYNC Symptom Questionnaire first hand from the patient in the consultation: ‘I find it hard to look at reports and make decisions. I like to see the patients in person, and that provides clinical context that is difficult to glean by reading something’ (A022). If the SYNC system could improve timeliness and efficiency of tests and treatment, then this may produce better outcomes for some patients but only if it does not delay their face-to-face consultation.

## Discussion

This qualitative study informs the design and contents of a patient-reported symptom-based risk stratification system prior to implementation of a new HNC diagnostic pathway for patients referred from primary care. The findings highlight symptomology and the language used by patients and clinicians to describe symptoms in urgent specialist appointments that will be important to consider when developing the SYNC Symptom Questionnaire. The findings also offer an in-depth understanding of the requirements for the SYNC system from the perspectives of patients and clinicians, key stakeholders in the pathway.

Patient groups, primary and secondary care doctors, national bodies and commissioners have called for the sHNC pathway to be redesigned,^19^ and a survey of 42 primary and secondary care professionals identified that referral information should be the focus of change.^20^ It can be difficult to identify high-risk HNCs in England due to poor referral information.^19^ This is important as HNCs can progress quickly—with even short delays in time to treatment being more likely to lead to reductions in survival than is the case for other common cancers such as breast and prostate.^21^ Clinicians interviewed in this study confirmed that referral information impedes effective and efficient triage in secondary care for patients with sHNC. They noted that terminology applied by GPs may make it hard to distinguish, for example, a lump in the throat from a palpable neck lump, an important distinction given all participating sites operated a specialist neck lump clinic. The HaNC-RC was developed for use by specialists to aid identification of high-risk patients.^10^ Our data demonstrate that it can require a series of questions for clinicians to generate the responses needed for completion of HaNC-RC and has highlighted the clarifications that may be needed to distinguish different symptoms. Using appropriate terminology and avoiding language more likely to be misinterpreted could enable patients to provide information about their symptoms at an earlier stage in their care pathway via a SYNC system, to support triage and allow for earlier investigation.

Previous research suggests that self-completed risk assessment tools are viewed positively by patients; two cluster randomised trials showed that these tools relieved patient anxiety, were associated with improved patient knowledge and had no adverse psychological effects.^22^ Patients interviewed in this study spoke of the potential for the SYNC system to relieve anxiety by giving the patient an active role in their care, where they can provide information about their symptoms during a time of inactivity while they are waiting for their appointment. This was not the view of all patients, however, with some feeling that a patient-facing symptom questionnaire could introduce anxiety. Patients proposed features that the SYNC system should include that may alleviate anxiety, for instance, links to in-person support from a health professional.

This study highlights the different ways in which patients and clinicians describe symptoms that may indicate HNC cancer and the techniques clinicians use to obtain the information they need from patients. It is vital that the SYNC Symptom Questionnaire uses language and terms that are meaningful to patients to enable them to report their symptoms accurately. As well as the accessibility of the language used, access to the online SYNC system itself was discussed by patients and clinicians. As of January 2024, internet access in the UK is high with 98% of the population having access.^23^ However, other factors including digital literacy, visual and cognitive impairments, learning difficulties, limitations with manual dexterity and a lack of confidence have been identified as impacting on peoples’ use of digital healthcare platforms.^24 25^ Our findings touch upon these accessibility issues and provide some potential solutions that may improve access to an online health system like the SYNC system and ensure patients’ emotional needs are supported. For example, patients suggested options for different modes of access to the SYNC system, such as a telephone call, and felt it was important to ensure that problems that could result from the remote nature of the online system are buffered by the availability of face-to-face care and support. The findings also have wider implications for understanding the impact of language on emotionally evocative healthcare interactions. The results show how clinicians use language and behaviour to build rapport with patients, provide reassurance and demonstrate empathy. Empathetic healthcare is associated with multiple benefits to patients.^26^ Research shows that it reduces patient stress and anxiety,^27^ enhances patient satisfaction and self-management and in turn can lead to improved clinical outcomes.^28^

A key strength of the study is the use of multiple qualitative methods to inform the development of a patient-reported symptom-based risk stratification system. Complimentary methods were used whereby observation of urgent specialist appointments and follow-up interviews with a subset of those patients and clinicians allowed the research team to gain an in-depth view of participant perspectives. The sites included in the study allowed for a broad mixture of social, economic and cultural backgrounds of potential participants. Recruitment information was available in different formats and languages to ensure inclusivity, and funding was available for translation services to include urgent specialist appointments with patients who do not speak English.

In terms of limitations of the study, it was disappointing and somewhat surprising that some sites were unable to provide ethnicity information about patients. Unfortunately, the sample of patients that were interviewed was not as diverse as intended. Although clinicians from different subspecialties were approached and invited for an interview, the responsiveness of clinicians to the study resulted in a sample of mostly ENT clinicians, with one oral maxillofacial clinician interviewed, limiting the generalisability of the findings. We had intended to conduct targeted recruitment of patients to ensure that recordings of consultations included the full range of symptoms included in the HaNC-RC-v2. However, information available at recruitment to inform patient selection varied in quality and quantity, and not all symptoms were captured. Only one observation of a clinic consultation was conducted due to practical reasons; however, this visit to site was positive for relationship building between the research team and the site and subsequent recruitment. The use of telephone interviews may have excluded participants without the technological facilities to take part, although we aimed to minimise this effect by using telephones rather than video-calling for patients, given that telephones are more widely used. Including the option for in-person interviews may have resulted in a sample of people with different perspectives than those reported, but unfortunately, this was not feasible due to the dispersed nature of the geographical locations in the study.

In conclusion, the current study explores the potential design of a novel patient-reported symptom-based risk stratification system for sHNC referrals. The findings offer in-depth insights about the language and terms that should be considered when designing a patient-facing symptom questionnaire that is understandable for patients and provides clinically useful information. Requirements of a patient-reported symptom-based risk stratification system, from the perspectives of those who will be the end users of the system, are also offered. Future research should extend the current study to explore the development of the SYNC system using a patient-centric co-design approach. It will be important to draw out the needs of different categories of user and refine our understanding of the contextual factors that will affect use of the SYNC system.

## supplementary material

10.1136/bmjopen-2024-094197 online supplemental file 1

10.1136/bmjopen-2024-094197 online supplemental file 2

10.1136/bmjopen-2024-094197 online supplemental file 3

10.1136/bmjopen-2024-094197 online supplemental file 4

## Data Availability

Data are available upon reasonable request.
