# Supplementary material for: Qualitative study to inform the design and contents of a patient-reported symptom-based risk stratification system for patients referred from primary care on a suspected head and neck cancer diagnostic pathway
Source: BMJ Open. 2025 Apr 3;15(4):e094197. doi: 10.1136/bmjopen-2024-094197 (PMC11969606; doi:10.1136/bmjopen-2024-094197)
Supplement: online supplemental file 1 [file bmjopen-15-4-s001.docx]

**Appendix A. Patient and clinician topic guides**

**EVEREST-HN**

**Using patient-reported symptoms to guide referral for suspected head and neck cancer**

**PATIENT INTERVIEW TOPIC GUIDE**

**Version 1.0**

**EVEREST-HN: Patient Interview Topic Guide**

*Note: The interview schedule is developmental. The questions will need to be tailored to the specific answers of each interviewee. The interview schedule given here is therefore a general topic guide for the one-to-one qualitative interviews.*

**Welcome and Introduction.**

Ask if any questions. Obtain informed consent

**Re-cap of Research and Plan for Interview**

Brief re-cap on the aims and purpose of the interview and explain what will happen.

***The following questions need not be covered in this particular order but rather the interview should flow as freely and naturally as possible. The interviewer will prompt as appropriate with phrases such as ‘can you tell me a little more about that’, ‘can you give me an example of that’, ‘how did/do you feel about that’.***

**Experience of cancer pathway**

It may be upsetting for the patient to talk about some parts of their experience. It is important to give them time to talk about this. Modify the questions about their pathway on the basis of what they say about their overall experience.

- **Can you tell me a bit about the symptoms that you have had in your head or neck and how you came to be referred for an urgent hospital appointment?**
  - Symptoms – how long, severity etc
  - Explore what they mean by terms “can you tell me a bit more about that?” “what do you mean by xxx?”
  - What prompted GP consultation (in some people it could have been picked up opportunistically at a routine dental appointment)
  - What did the GP say to you about why they were referring you to hospital?
  - How did you organise the time to go to the hospital?
    - Choice?
- **How did you feel about being referred for an urgent hospital appointment?**
  - Pleased, anxious?
- **How did you find the period while you were waiting for the hospital appointment?**
  - Anxious?
  - Information searching?
- **Can you tell me what happened at your hospital appointment?**
  - Who was there?
  - Tests etc
- **What has happened since that first hospital appointment?**
  - Tests

**The EVEREST study is aiming to improve the urgent cancer referral pathway. It would be good to know from your point of view what was good about your experience and what could be improved.**

- **What things were good about your experience of an urgent referral?**
- **What things could have been better?**

**EVEREST**

**The next questions are about one idea to improve the urgent cancer referral pathway for patients. We would like to hear what you think of it, as someone who has been through an urgent cancer referral recently.**

**The idea is that patients would be asked some questions about their symptoms either on a computer or on the phone. This would be in the time between seeing the GP (or dentist) and going to the hospital. The questions might be about symptoms like a sore throat, a blocked nose or a lump in your neck. The answers would be given to the hospital doctors to look at before they see the patients. Depending on the answers they give, some people might be sent for tests straight away rather than waiting to see a doctor first.**

**Thinking about people like you who have an urgent referral, how do you think people would feel if they were asked to answer some questions about their symptoms on a computer or on the phone?**

- Do you use a computer/smart phone?
- Do you ever use a computer or phone for things like shopping, booking tickets or checking health information (e.g. NHS 111)?
- How do you feel about using the computer for things like that?
  - Convenient or hard work/stressful?
  - Are there any websites that you can think of that are particularly bad or good?
- How do you feel about the idea of answering questions from the hospital about your health on a computer or phone?
- Do you think it would have made you feel more or less anxious to be asked to answer some questions?
- Is there anything that you think might help patients to answer the questions?
- How do you feel about the idea that a computer might help decide whether you were sent straight for tests before you saw a doctor?
  - Do you think it is ok for a computer to help with those decisions or do you think it should always be the doctor?
    - What if the computer made it quicker to get an answer about whether you have cancer or not?

**I’d like to ask you about being invited to take part in the EVEREST-HN study. Can you tell me how you were first informed about the study?**

- Who was there?
- What else was going on in the appointment?
- Can you remember what they said?
- Can you remember what your first reaction was?

**How did you decide whether to take part in the study?**

- How did you make your decision?
- What things were important to you?
- Did you speak to other people about the decision? If so, who?
- When did you make the decision?
  - Immediately/nurse phone call/recruitment discussion
- Did you find it an easy decision to make? Why (not)?

**Do you remember being given some written information about the study?**

- What did you think about that information?
- Was it helpful?
- How did you use it?
- Do you have any suggestions for how it could be improved? If so, how?

**Would you have liked information about anything else?**

- If so, what?
- Why was that important to you?

**Anything Not Covered?**

Is there anything that we haven’t covered in the interview that you think we should know or think about?

**Closing and Thanks**

Conclude the discussion and thank the participant for their time and contribution.

**EVEREST-HN**

**Using patient-reported symptoms to guide referral for suspected head and neck cancer**

**STAFF INTERVIEW TOPIC GUIDE**

**Version 1.0**

**EVEREST-HN: Staff Interview Topic Guide**

*Note: The interview schedule is developmental. The questions will need to be tailored to the specific answers of each interviewee. The interview schedule given here is therefore a general topic guide for the one-to-one qualitative interviews.*

**Welcome and Introduction.**

Ask if any questions. Obtain informed consent

**Re-cap of Research and Plan for Interview**

Brief re-cap on the aims and purpose of the interview and explain what will happen.

***The following questions need not be covered in this particular order but rather the interview should flow as freely and naturally as possible. The interviewer will prompt as appropriate with phrases such as ‘can you tell me a little more about that’, ‘can you give me an example of that’, ‘how did/do you feel about that’.***

**Cancer diagnostic pathway**

Initial questions about the referral pathway at the specific trust may not be needed if they have been covered in previous interviews.

**Can you tell me about how the head and neck urgent cancer referral pathway is organised at the Trust?**

- - ***Establish in detail (who (including involvement of Nurses and AHPs), how, when):***
    - ***how referrals are processed***
    - ***how clinics are organised***
    - ***how onward referrals are made***

**I’d like to know about changes that happened in the referral pathway during COVID-19 – how did you manage urgent referrals during that period?**

- - ***Did they use any means of triaging referrals? – if so find out about this in detail and about their experiences of this.***

**What do you think about the recent changes in targets for cancer diagnosis? Are you aware of any changes at your hospital as a result of the new targets?**

**Can you tell me about your role within the head and neck urgent referral cancer pathway?**

The EVEREST study is aiming to improve the diagnostic pathway for urgent head and neck cancer referrals. It would be good to know from your point of view what is good about the current pathway and what could be improved.

- **What things do you think are good about the current diagnostic pathway for urgent referral?**
- **What things could be better?**
- **Are there particular sub-groups of patients that are more straightforward or harder to assess?**

**You probably remember that we observed/recorded your consultation with (name). Would it be ok if we talked a bit about the consultation so that we can understand more about the processes that were happening?**

***Specific questions about e.g. why they pursued a particular line of questioning, or what triggered a decision to request a particular test***

The EVEREST study is planning to introduce an intervention within the diagnostic pathway. Patients will be asked some questions about their symptoms either on a computer or on the phone. This would be triggered by the receipt of the referral at the hospital. Their answers would be used to calculate a risk score which would be made available to the hospital team, along with any additional information provided by the patient. We hope that this might enable the hospital team to make some decisions ahead of a hospital appointment, e.g. about tests or who needs to be seen most quickly and to provide relevant information in a standardised and accessible format.

**What do you think of this idea?**

- ***Feasible? (digital exclusion)***
- ***Useful? (an improvement? Would they trust a risk score?)***
- ***How would you see it working? (who might be involved – what roles might change? Who would need to be involved in planning for change?)***
- ***Add to or reduce workloads?***
- ***Is this the best way to improve the pathway or is there something that you think would work better?***

**Anything Not Covered?**

Is there anything that we haven’t covered in the interview that you think we should know or think about?

**Closing and Thanks**

Conclude the discussion and thank the participant for their time and contribution.
