# Supplementary material for: Qualitative study to inform the design and contents of a patient-reported symptom-based risk stratification system for patients referred from primary care on a suspected head and neck cancer diagnostic pathway
Source: BMJ Open. 2025 Apr 3;15(4):e094197. doi: 10.1136/bmjopen-2024-094197 (PMC11969606; doi:10.1136/bmjopen-2024-094197)
Supplement: online supplemental file 2 [file bmjopen-15-4-s002.docx]

Appendix B: Template Rapid Assessment Procedure (RAP) sheets for interviews and consultations

**EVEREST-HN WP1**

**RAP sheet for clinician interviews**

Patient identifier:

Clinician identifier:

| **Topic** | **Descriptive information from transcript** | **How this integrates in to developing analysis** |
| --- | --- | --- |
| Interview summary |  |  |
| Organisation of current HNC urgent referral pathway at the trust |  |  |
| Changes during Covid-19 pandemic |  |  |
| Role of clinician in the pathway, including triage, questions asked/how asked/language used, how reassures and gives info to patients. |  |  |
| Perspectives on current pathway for instance what worked well and what they’d like to change or see improved |  |  |
| Clinician feedback on SYNC idea |  |  |
| Any other information, including about research process |  |  |

| **EVEREST-HN WP1**  **RAP sheet for patient interviews**  Patient identifier:  Clinician identifier: | | |
| --- | --- | --- |
| **Topic** | **Descriptive information from transcript** | **How this integrates in to developing analysis** |
| Interview summary |  |  |
| Terms used by patient to describe symptoms |  |  |
| Language used by clinicians or others that patient finds helpful, e.g.:   - Clarifying - Information-giving - Reassuring - Rapport building (not just language but tone of voice) |  |  |
| Terms that patients describe as difficult to understand or upsetting/negative and ideas about how to communicate more clearly/positively |  |  |
| Patient personas- characteristics of patients and links to terms they use/understand and poorly understood terms. |  |  |
| Perspectives on current pathway for instance what worked well and what they’d like to change or see improved |  |  |
| Patient feedback on SYNC idea |  |  |
| Any other information, including about research process |  |  |

| **EVEREST-HN WP1**  **RAP sheet for consultations**  Patient identifier:  Clinician identifier: | | |
| --- | --- | --- |
| **Topic** | **Descriptive information from transcript** | **How this integrates in to developing analysis** |
| Consultation summary |  |  |
| Terms used by patients to describe symptoms  “White stuff going on in the back of my throat”; “My voice just changed”; “Sore throat”; “Swellings on the neck” |  |  |
| Language used by clinicians that’s helpful in generating relevant responses from patients  **Clarifying** – going back over what’s been said, paraphrasing to check understanding, step-by-step approach.  **Information-giving** – providing clear information about treatment.  **Reassuring** – “We don’t actually need to do anything about this at all”. Sometimes patients cannot be reassured in the moment.  **Rapport building** – using patient’s name, humour, chat, empathy.  Not just language but **tone of voice** – even, professional, warm but not emotional.  **New category- maintaining focus-** describes the techniques clinicians use to keep consultation on track and obtain relevant information |  |  |
| Terms that appear to be poorly understood by patients and how clinicians communicate complicated terms  When patients don’t seem to understand, clinicians explain – e.g. translate ‘GA’ to ‘general anaesthetic’.  Often use medical term and then explain, rather than ‘translate’ completely.  Cultural differences – patients says has never smoked but it emerges that he chews paan. |  |  |
| Patient personas- characteristics of patients and links to terms they use/understand and poorly understood terms. |  |  |
| Structure and flow of consultation/order of events. |  |  |
| HaNC-RC question areas (below; how are these asked about and followed-up in consultation):   - Unintentional weight loss - Smoking status - Alcohol status - Hoarse voice - Sore throat - Difficulty swallowing (dysphagia) - New neck lump - Pain on swallowing (Odynophagia) - Oral ulcer - Oral swelling - Unilateral ear pain with normal ear examination - Noisy breathing (Stridor) - Persistent head and neck skin lesion - Feeling of something/lump in throat |  |  |
| Use of existing information/evidence about the patient in consultation eg. Information from GP, previous tests. |  |  |
| Questions asked by clinician that are not commonly asked during other consultations/by other clinicians |  |  |
| General comments |  |  |
