# Supplementary material for: Qualitative study to inform the design and contents of a patient-reported symptom-based risk stratification system for patients referred from primary care on a suspected head and neck cancer diagnostic pathway
Source: BMJ Open. 2025 Apr 3;15(4):e094197. doi: 10.1136/bmjopen-2024-094197 (PMC11969606; doi:10.1136/bmjopen-2024-094197)
Supplement: online supplemental file 3 [file bmjopen-15-4-s003.docx]

| **Appendix C. Supplemental Table 1. Suspected head and neck cancer symptomology and language used by patients and clinicians to describe symptoms – additional exemplars.** | | |
| --- | --- | --- |
| **HaNC-RC-v2 items** | **Examples of questions asked by clinicians during consultations to understand patient lifestyle and elicit symptoms** | **Examples of terms used by patients to describe their lifestyle and symptoms during consultations** |
| **Unintentional weight loss** (Yes/no) | ‘Have you lost weight?’ Clinician asks about timeframe for weight loss ‘In space of what?’ (B013).  ‘Have you lost any weight unintentionally since it’s [symptoms] come on? (B001).  Have you had any weight loss recently? Are you *trying* to lose weight? (Patient has lost weight and is not trying to but said he was overweight before. Clinician goes on to ask further questions). How much weight loss have you noticed? How long has that been? How’s your energy level? (A037).  Did you lose that weight on purpose? (A005)  You haven’t lost any weight? (B019)  Your weight is stable? You’re not losing weight? (B013)  Have you lost any weight? Is your appetite good? (B002) | When asked if he’d lost weight intentionally, patient B023 and girlfriend laugh and he says ‘nooo, I’ve put it on!’  Yes I am losing a bit of weight which is a good thing as I have been overweight. My waist has gone from a 40 to a 38. (A040).  Nah, I wish I did. Doctor keeps saying I got to lose it for the knee operation but that’s not happening at the moment is it! (B032)  When patient went to the doctor last week ‘she said she lost a little bit, not a lot’ (Patient’s daughter interrupts to give this information) (B010). |
| **Smoking status** (never smoked, ex-smoker, current smoker) | Do you smoke? Patient didn’t smoke and clinician doesn’t ask if patient had ever smoked (B013).  Do you smoke? Have you ever smoked? When you did smoke, how often? (B009)  Ever been a regular smoker? (B001)  At the peak how many a day did you smoke? (A009)  Have you ever smoked? Do you currently smoke? How many a day would you have smoked, at the peak? For how many years did you smoke? (A009)  Do you smoke? Ever smoked? And you do not use tobacco? (B002)  ‘Are you or were you ever a smoker?’ ‘No, not a smoker’. Later, when examining mouth sees staining and asks: ‘Have you ever chewed paan and do you still chew?’ Yes. (B001, patient B007).  Patient says he uses/used (can’t tell on recording) tobacco and B019 asks ‘betel nut?’ Silence. ‘Paan’ and patient confirms. B019 asks ‘Are you still using it?’ Patient answers ambiguously and then says he still uses it but not as much. B019 gives him options: Five? A day? Yes. In the past: more? Yes. (B019, patient B031). | Patient (B017) talks about his father being a heavy smoker and mentions ‘passive smoke as a child…’ and clinician B001 interrupts and clarifies: were you ever a smoker? Patient replies no.  Patient (A040) says no, I did [smoke] years ago but I don’t now. Clinician (A037) asks when did you stop smoking? Erm 40 years ago.  Clinician (B002) tells patient ‘half of the problem [symptoms] is smoking related’. Patient responds ‘I’ve got to the point where I’ve decided if it’s affecting my health then I will pack up and I’ve got the determination to do it’. (B045) |
| **Alcohol status** (≤14 units/week, >14 units/week, ex-excess) | Clinician just asks ‘alcohol?’. Patient says only if she goes out and clinician says ‘minimal’. (B013).  Do you drink any alcohol? How many units would you say you drink in a week? (B009).  Ever been a big drinker? (A005)  Do you drink much alcohol? (B001).  Alcohol, do you take much? How much do you take on an average week? (A037)  How much do you drink in a week? (A009) | Patient B023 says he drinks at the weekend and girlfriend echoes this. He says 4 units ‘whatever the doctor said, it was 4 units’- patient mentions units without being prompted.  Patient (A041) estimates how much she drinks ‘about 4 bacardi’s on a Friday and Saturday night’. Clinician misinterprets and says ‘so 4 Bacardi’s a week’- patient replies ‘no on a Friday and Saturday so that would be 8..’. Patient jokes ‘go on put 10 to be a devil’.  I don’t know units wise (clinician asks ‘just in general?’). I’d say two bottles of wine and a couple of lagers [per week]. (A044)  In units, about 15. Clinician (A009) says doesn’t have to be units; what you think. '6-7 glasses of beer a week and a couple of glasses of wine; Not that much’. (A043)  I have the occasional glass of wine (B043)  I drink a bottle of wine at the weekend, that’s it (C017) |
| **Hoarse voice** (no, intermittent, persistent explained, persistent unexplained) | ‘When did you first notice problem with your voice?’ ‘How long have you had this for?’ ‘Do you lose your voice completely?’ ‘So, once it comes, how long does it take to improve?’ (B013).  In terms of the voice, do you think it’s different?’ ‘Is it more hoarse?’ ‘Over what period do you think?’ (B009).  Can you point with one finger to where that (discomfort) is or is it generalised? Clinician says ‘Your voice fluctuates (this is a positive sign). (B001)  Your voice sounds ok. Is that normal for you?’ (B001)  Any change in your voice? (A009)  What you’re bringing up, is it mucky at all? (C006) | It was a *wheezy* cough but now it’s a really… strange sort of cough and I can wake up in the night, and I’m choking, it’s as if I’ve had a drink. (B035).  Patient describes voice as a bit ‘gravelly’. (B034).  My voice feels hoarse if I’ve been talking much, you can hear it a bit now. (B022)  I normally lose it [voice] totally, it goes all squeaky... it comes back a couple of weeks later (B032)  It’s a bit scratchy (A007)  I’ve been coughing and my voice has been coming and going and some days I couldn’t speak…I think it’s mucus (C050)  My voice feels deeper and weak (C053) |
| **Sore throat** (no, unilateral intermittent, bilateral/midline intermittent, unilateral persistent, bilateral/midline persistent) | Does your throat become sore after prolonged use [of voice]? (B001)  Do you get a sore throat for a long period of time? (A037)  Which side is the soreness? Did it start with a cold? When did you have the dental treatment? (A005)  Do you have a constant desire to clear your throat? Do you have a dry mouth in the night? (B002) | A bit of sharpness but just on one side (A045)  It’s making my throat sore, very sore and I’m often losing my voice (B032) |
| **Difficulty swallowing** (dysphagia) (No, intermittent, persistent) | Do you actually have any difficulty in swallowing?’ (B001)  Do you have any food stick? (A005)  Do you think things sometimes go down the wrong way? (C013)  Are you eating and drinking ok? As in, swallowing ok? (C006)  Can you eat and drink whatever you want? (C013)  Do you feel like you’re trying to clear your throat a lot? Like *makes throat clearing sound*, as if you’re trying to get rid of something? (C006) | It’s sore, and when I swallow.. you know, like a muffin, I can’t even swallow that. It seems as if it gets stuck *there*. I always need to drink. (B035).  When I’m swallowing, it almost gets a little bit tighter and it’s almost hard to clear my throat properly. I feel like I’ve got something stuck in there’. (A025)  I’m eating and then I swallow and then I have to cough to sort of dislodge it or drink some fluid to make it go down’. (A025)  Occasional cough as if I’ve swallowed something the wrong way. (B022)  The food goes down but it’s just like a spasm (A043)  Something is getting stuck and having to wait for it to go down (B039) |
| **New neck lump** (No, fluctuating/reducing, persistent) | I know you’ve got a sensation of something but have you ever actually felt a lump with your fingers? (B001)  Have you had any glands come up that you can feel? (A005)  Has it changed in size at all? Does it hurt? Have you had any redness over the skin on the lump? (B009)  When did you first notice the neck lump and has it got any bigger since then? Does the lump change when you eat and drink? (A005)  Any lumps or bumps anywhere else? (C006) | Pressure as if it was swollen on my neck. (B022)  A few times I’ve felt a wave of pain inside [the lump]..something like a pinch [in the lump]. (A017)  You get paranoid with every little lump and bump…it feels symmetrical but tender (A045)  I’ve been getting a swelled throat, but that last time it happened it was massive, like my neck…there was no jaw, everything was just neck (C044) |
| **Pain on swallowing** (Odynophagia) (Yes/no) | ‘Is it painful when you swallow?’ ‘Would you describe it as discomfort?’ Clinician checks which side: ‘It’s only on the right? (B009).  Do you have pain on swallowing? (A037)  Is it tender when you swallow? (A005) | Patients generally give short answer either yes or no |
| **Oral ulcer/ oral swelling** (Yes/no) | Do you get tonsil stones very often? (A037)  Are the ulcers painful? How long did pain last for? How long did ulcers last for? Did they reoccurr in a different site? Does it bleed? Does it affect your speech? Anything that would have made the ulcers reoccur or worse, any food or anything you’re allergic to? (A019)  When you start eating, do you get any swelling on your neck or any pain? (A019) | ‘I had a hole at the side of my tonsilitis.. I thought it was infected, I thought it was tonsillitis’ (A039)  There’s a cut on my tongue, on the side of my tongue that’s been there for a while (B032)  I had a small ulcer on the front of my tongue, very painful (A045)  There was a swelling and then it went (A045)  I got a whole bunch of [mouth ulcers] on the bottom of my tongue and it was so painful, I couldn’t eat, talk or anything (A020).  I feel like I’ve got a bit of a lump in the side of my face, here *points* (A030)  Tingling and numbness in my bottom lip (B045)  I noticed an ulcer type thing on my tongue (B039) |
| **Unilateral ear pain with normal ear examination** (Yes/no) | ‘Have you had any pain in the ears? Which side?’ So you would say you’ve got some right-sided ear pain?’ ‘Is that on and off or constant?’ (B009)  Have you had any earache? Do you get it most days or just occasionally? (A005)  Are you getting any shooting pains in the ear when you swallow? (C013) | Patient describes ear pain as ‘jabbing’. (B035).  My ears are blocked. (A034)  My ears are itchy sometimes (B041) |
| **Noisy breathing (Stridor)** (Yes/no) | No examples in consultations | No examples in consultations |
| **Persistent head and neck skin lesion** (Yes/no) | No examples in consultations | No examples in consultations |
| **Feeling of something/lump in throat** (Yes/no) | Apart from the cough, do you have any pain in your throat? Do you have any trouble eating or drinking? (A037)  Is it [lump in throat] worsening or the same? (B002)  Has it changed in a year? (C013) | I’m drinking a lot of tea to flush it down. I have a job to swallow. (A040)  I could feel a lump on the inside of my throat (B045)  When I’ve had my dinner, a couple of hours later, if I cough, there’s like bits of food still there (C014) |
