# Supplementary material for: Qualitative study to inform the design and contents of a patient-reported symptom-based risk stratification system for patients referred from primary care on a suspected head and neck cancer diagnostic pathway
Source: BMJ Open. 2025 Apr 3;15(4):e094197. doi: 10.1136/bmjopen-2024-094197 (PMC11969606; doi:10.1136/bmjopen-2024-094197)
Supplement: online supplemental file 4 [file bmjopen-15-4-s004.docx]

| **Key requirement** | **Exemplary quote** |
| --- | --- |
| **Patient perspective** | |
| Patient data stored securely | ‘What I would be bothered about is the security of the information *[given when completing SYNC]*’ (C043). |
| Availability of different methods to access and complete SYNC | You need failsafes for people so they are not disadvantaged by the format. An initial question would be, do does this *[completing SYNC]* by computer make you anxious or is difficult I anyway? If so, do you want to opt for a telephone conversation?’ (B044) |
| Must capture all patient symptoms | ‘A questionnaire is not all encompassing and not everyone fits in to that box’ (C043). |
| Sources of in-person support | ‘It would be nice to have a *live* person to talk to *[when answering SYNC]*’ (B022). |
| Subsequent in-person appointment | ‘As long as you later get seen by somebody *[a health professional].* When you come to this point that you are so concerned that you’re seeing your GP and the GP has referred you, you want like an answer’ (C022). |
| **Clinician perspective** | |
| Evidence-based risk stratification | ‘As long as it’s validated and it’s helping to stratify risk then that would be helpful’ (A022). |
| Effective discrimination of symptoms | ‘If we can make our one-stop clinic fully one-stop and this triage can support that by the appropriate wording of questions, that would be important. And I think wording cannot be underestimated. And a good example of that in this process is how GPs will tick a neck lump box on a referral form very freely. But that form does not discriminate whether there is a visible or feelable, palpable neck lump’ (B001). |
| Automated system | ‘It *[SYNC]* must be automated before the patient comes in clinic as to not increase clinician workload’ (A004). |

**Appendix D Supplemental Table 2: Key requirements for a patient-reported symptom-based risk stratification system for sHNC**
