# Supplementary material for: Qualitative study to inform the design and contents of a patient-reported symptom-based risk stratification system for patients referred from primary care on a suspected head and neck cancer diagnostic pathway
Source: BMJ Open. 2025 Apr 3;15(4):e094197. doi: 10.1136/bmjopen-2024-094197 (PMC11969606; doi:10.1136/bmjopen-2024-094197)
Supplement: Supplementary file 5 [file bmjopen-2024-094197.reviewer_comments.pdf]

PEER REVIEW HISTORY

BMJ Open publishes all reviews undertaken for accepted manuscripts. Reviewers are asked to complete a checklist review form and are provided with free text boxes to elaborate on their assessment. These free text comments are reproduced below.

ARTICLE DETAILS

Title (Provisional)

A qualitative study to inform the design and contents of a patient-reported symptom-based risk stratification system for patients referred from primary care on a suspected head and neck cancer diagnostic pathway

Authors

Albutt, Abigail; McVey, Lynn; Randell, Rebecca; Hardman, John C; Kellar, Ian; Odo, Chinasa; Patterson, Jo; Bradley, Paula; Davies, Claire; Tikka, Theofano; Paleri, Vinidh; Rousseau, Nikki

---

VERSION 1 - REVIEW

**Reviewer**	**1**
**Name**	**Wei, Xing**
**Affiliation**	**Sichuan Cancer Hospital and Research Institute, Department of Thoracic Surgery**
**Date**	**17-Nov-2024**
**COI**	**None**

Comment to authors:

The manuscript presents a well-designed and comprehensive qualitative study that explores patient and clinician perspectives on the proposed SYNC system for patient-reported symptom-based risk stratification in the head and neck cancer referral pathway. By utilizing multiple data sources, including clinic observations and interviews with both clinicians and patients, and being guided by clearly defined research objectives, the study demonstrates its relevance in enhancing triage processes, clinical decision-making, and patient outcomes within the cancer care pathway. This work holds significant implications for advancing patient-centered care.

Below are my specific comments and recommendations:

1. The Methods section, while descriptive of various data collection methods such as observations and interviews, appears somewhat repetitive and lacks coherence. The inclusion of a "Methods" subheading within the "Methods and Analysis" section contributes to structural confusion. Reorganizing this section to eliminate redundant headings and presenting content in a logical sequence—grouped by data collection, data analysis, and study design—would improve clarity and reader comprehension.

2. Key terms, such as "risk stratification" and "patient-reported symptoms," should be consistently defined and used throughout the manuscript. This would facilitate precise understanding and prevent ambiguity. Conducting a thorough editorial review to ensure consistent terminology and expressions would further enhance the overall readability and coherence of the manuscript.

3. In Table 1, the authors have used the term "gender." When discussing biological factors, the term "sex" should be used, whereas "gender" is appropriate for discussing gender identity or psychosocial/cultural factors. It is recommended to replace "gender" with "sex" to ensure accurate terminology and alignment with best practices.

4. The manuscript is quite lengthy; it would be beneficial to consider streamlining the content, focusing on the core findings and key points to enhance the conciseness and readability of the article.

Reviewer **2**

Name **Wan, Hongwei**

Affiliation **Shanghai Key Laboratory of Radiation Oncology, nursing Department of Nursing**

Date **02-Jan-2025**

COI **None**

This is an interesting study that provides insights into the development of symptom checklists for patients with head and neck cancer before referral from primary care to new diagnostic pathways, and outlines the requirements for a patient-reported symptom-based risk stratification system. However, the scope of the primary research question is unclear, and the study objectives and extracted qualitative themes seem unrelated to the research question stated in the title. The research methodology and design lack scientific rigor. Furthermore, as a qualitative study, the writing is not sufficiently scientific and contains numerous issues. Below are some specific concerns:

1. The title is overly lengthy and resembles the research objectives of the study; it is recommended to simplify the title to conform to conventional standards for research titles.

2.Page 2 Introduction

“Given the rarity of HNC, there is limited experience within primary care for HNC diagnosis; however, the symptoms associated with sHNC (e.g. a feeling of a lump in the throat) are common, and thus large numbers are referred for specialist opinion into secondary care services, which must be configured to meet this demand.” Head and neck cancer is already the sixth most common malignancy worldwide, and its referral from primary care is not due to its rarity. It is generally considered that the associated symptoms are common but can easily be confused with those of other diseases, thus necessitating referral. This part of the description is not adequately justified.

“Head and Neck Cancer Risk Calculator(HaNC-RC) symptom inventory” The content of the checklist and the associated issues are unclear.

“The evolution of a patient-reported symptom-based risk stratification system to redesign the suspected head and neck cancer referral pathway (EVEREST- HN) Programme proposes a novel approach to redesign the sHNC pathway that may enable earlier diagnosis of cancer, or earlier reassurance that the patient is cancer free.”There is a lack of reasonable explanation or mechanism regarding how the patient-reported symptom-based risk stratification system could improve referral practices for head and neck cancer patients. The specific details of the risk stratification system are also unclear, and it remains uncertain whether the system can effectively perform risk stratification.

Which populations will be interviewed in the qualitative study? What is the expected content of the interviews? The objectives, target population, and purpose of the qualitative study are not clearly outlined in the study background.

3.Page 3 Aims and objectives: “The study objectives are to explore: • how clinicians ask questions and decide subsequent steps for patients referred with sHNC; • the language patients and clinicians use to describe symptoms; • how clinicians reassure and discharge low-risk patients; and • clinician and patient experiences of the current diagnostic pathway and their views on the novel EVEREST-HN pathway.” The research objectives are unrelated to the theme stated in the title, which suggests providing information on the design and content of a patient-reported symptom-based risk stratification system.

4.Page 3 "Design": There is a lack of description of qualitative research methods. What is the role of using multiple qualitative methods in this study? Why are multiple qualitative analysis methods employed?

5.Page 3 "Participants and Setting": The inclusion and exclusion criteria for patients are missing.

6.Page 3 "Data Collection": The data collection methods and specific procedures are not provided.

7. Page 5 "Results": There is an absence of a table describing the general demographic information of the patients. Additionally, the rationale behind the use of maximum variation sampling for selecting study participants needs to be explained.

8.Page 5 “Optimising a patient-reported symptom inventory for HNC ”， Page7“Patient and clinician interviews: Requirements for a patient-facing Symptom iNput Clinical (SYNC) system”The extracted themes from the qualitative research results appear unrelated to the research topic, and subthemes under each theme are missing. The extraction of themes in qualitative research lacks logical coherence. The presentation of research findings should not consist of merely qualitative themes with one or two sentences describing or introducing each theme. The description of each theme should be a layered, progressive process.

9.A summary table of themes, subthemes, and patient quotations/examples is missing.

10.Page 9: The discussion section is insufficiently structured. It should focus on discussing the differences in the previous symptom checklists used in the risk stratification system and the reasons for providing the information.

VERSION 1 - AUTHOR RESPONSE

**Reviewer 1**	
The Methods section, while descriptive of various data collection methods such as observations and interviews, appears somewhat repetitive and lacks coherence. The inclusion of a "Methods" subheading within the "Methods and Analysis" section contributes to structural confusion. Reorganizing this section to eliminate redundant headings and presenting content in a logical sequence—grouped by data collection, data analysis, and study design—would improve clarity and reader comprehension.	Thank you for your comment. We have reduced the number of headings and used the headings suggested to organise the methods section. We hope this has improved readability.
Key terms, such as "risk stratification" and "patient-reported symptoms," should be consistently defined and used throughout the manuscript. This would facilitate precise understanding and prevent ambiguity. Conducting a thorough editorial review to ensure consistent terminology and expressions would further enhance the overall readability and coherence of the manuscript.	Thank you. We have been through the paper and hope that we have now provided clear and consistent use of these terms.
In Table 1, the authors have used the term "gender." When discussing biological factors, the term "sex" should be used, whereas "gender" is appropriate for discussing gender identity or psychosocial/cultural factors. It is recommended to replace "gender" with "sex"	Gender has been changed to sex in the table. Thank you for pointing this out.

to ensure accurate terminology and alignment with best practices.	
The manuscript is quite lengthy; it would be beneficial to consider streamlining the content, focusing on the core findings and key points to enhance the conciseness and readability of the article.	We have reworded parts of the findings to improve conciseness and draw out the key findings. We hope this enhances the readability of the paper.
Reviewer 2	
1. The title is overly lengthy and resembles the research objectives of the study; it is recommended to simplify the title to conform to conventional standards for research titles. [NOTE FROM THE EDITORS: Please feel free to rebut this comment - BMJ Open favours article titles that explicitly indicate the aim or the study or research question]	We have reworded the title which is now slightly shorter.
2.Page 2 Introduction<br>"Given the rarity of HNC, there is limited experience within primary care for HNC diagnosis; however, the symptoms associated with sHNC (e.g. a feeling of a lump in the throat) are common, and thus large numbers are referred for specialist opinion into secondary care services, which must be configured to meet this demand." Head and neck cancer is already the sixth most common malignancy worldwide, and its referral from primary care is not due to its rarity. It is generally considered that the associated symptoms are common but can easily be confused with those of other diseases, thus necessitating referral. This part of the description is not adequately justified.<br><br>"Head and Neck Cancer Risk Calculator(HaNC-RC) symptom inventory" The content of the checklist and the associated issues are unclear.<br><br>"The evolution of a patient-reported symptom-based risk stratification system to redesign the suspected head and neck cancer referral pathway (EVEREST- HN) Programme proposes a novel approach to redesign the sHNC pathway that may enable earlier diagnosis of cancer, or earlier reassurance that the patient is cancer free."There is a lack of reasonable explanation or mechanism regarding how the patient-reported symptom-based risk stratification	Thank you, we have amended this text which now says:<br><br>*"The symptoms associated with sHNC (e.g. a feeling of a lump in the throat) are common and can be difficult to distinguish from other conditions. As a result, large numbers of patients with sHNC are referred from primary care for specialist opinion into secondary care services, which must be configured to meet this demand."*<br><br>We have now added a cross reference from the mention of HaNC-RC to table 2 where items from the checklist are included<br><br>We have added content to the section where we outline the proposed mechanism for the risk-stratification system. This now reads:<br><br>*Implementation of SYNC could permit higher-risk patients to be prioritised, targeted investigations to be arranged directly before patients are seen in clinic, and potentially enable assessment for lower risk patients via alternative pathways that might be better able*

system could improve referral practices for head and neck cancer patients. The specific details of the risk stratification system are also unclear, and it remains uncertain whether the system can effectively perform risk stratification.<br><br>Which populations will be interviewed in the qualitative study? What is the expected content of the interviews? The objectives, target population, and purpose of the qualitative study are not clearly outlined in the study background.	*to address symptom management (e.g. speech and language therapy – led clinics).*<br><br>We agree that it is important to assess whether the system can effectively perform risk stratification. The work reported in this paper is EVEREST WP1. Later WPs within the EVEREST programme will assess first the feasibility and acceptability of the intervention and then if deemed acceptable will progress to a full cluster randomised controlled trial of effectiveness.<br><br>We have added details about the objectives and purpose of the qualitative interviews in the methods on pages 3 and 4 in the study design and data collection sections and highlight information that was in the original submission. See below for text.<br><br>*A sub-set of the patients and clinicians were purposively sampled from the wider sample to participate in a qualitative interview. Purposive sampling was based on demographic information including age, ethnicity, socioeconomic status and presenting symptoms, and to ensure each study site was represented.*<br><br>*Patients were invited to discuss their recent experience of the current diagnostic pathway and their views on the proposed SYNC system. Clinician interviews focused on the current diagnostic pathway, clinical decision-making processes prior to and during consultations and their views on the proposed SYNC system.*
3.Page 3 Aims and objectives: "The study objectives are to explore: • how clinicians ask questions and decide subsequent steps for patients referred with sHNC; • the language patients and clinicians use to describe symptoms; • how clinicians reassure and discharge low-risk patients; and • clinician and patient experiences of the current diagnostic pathway and their views on the novel EVEREST-HN pathway." The research objectives are unrelated to the theme stated in the title, which suggests providing information on the design and content of a patient-reported symptom-based risk stratification system.	Thank you for identifying that we had not communicated our objectives clearly.<br>The aim of this work is to **inform** the design and content of a patient-reported symptom-based risk stratification system. The work did this by identifying key considerations for the design of the system and by identifying suitable language (design and development was conducted in a separate work package which followed the work reported in this paper). We have added an additional objective to clarify this within the aims and objectives section:<br><br>*"2. To use information from objectives 1a)-d) to outline key requirements and appropriate*

	language for use in a SYNC system and SYNC Symptom Questionnaire."
4.Page 3 "Design": There is a lack of description of qualitative research methods. What is the role of using multiple qualitative methods in this study? Why are multiple qualitative analysis methods employed?	Detail about the use of multiple qualitative methods in the study has been added to the design section. See below for text.<br><br>*Multiple qualitative data collection methods were used to develop a comprehensive understanding of current practice within the sHNC pathway at the trusts (using interviews, observations and recordings of clinic consultations), the language used during consultations (recordings of clinic consultations) and the context within which care is delivered (interviews with key stakeholders).*
5.Page 3 "Participants and Setting": The inclusion and exclusion criteria for patients are missing.	Inclusion/exclusion criteria for patients has been added to study design section. Thank you for pointing out this omission.
6.Page 3 "Data Collection": The data collection methods and specific procedures are not provided.	We have outlined the methods used on page 3 under the data collection heading. See below.<br><br>*Data collection comprised of non-participant observation and audio-recordings of diagnostic consultations, patient interviews, and clinician interviews.*
7. Page 5 "Results": There is an absence of a table describing the general demographic information of the patients. Additionally, the rationale behind the use of maximum variation sampling for selecting study participants needs to be explained.	Demographic information can be found in Table 1 on pages 5-6. Our purposive sampling criteria is now described:<br><br>*Purposive sampling was based on demographic information including age, ethnicity, socioeconomic status and presenting symptoms, and to ensure each study site was represented.*
8.Page 5 "Optimising a patient-reported symptom inventory for HNC ", Page7"Patient and clinician interviews: Requirements for a patient-facing Symptom iNput Clinical (SYNC) system"The extracted themes from the qualitative research results appear unrelated to the research topic, and subthemes under each theme are missing. The extraction of themes in qualitative research lacks logical coherence. The presentation of research findings should not consist of merely qualitative themes with one or two sentences describing or introducing each theme. The description of each theme should be a layered, progressive process.	The aim of the current research was not to generate themes but rather key requirements and suggested language to inform the design and development of the SYNC system. As such we used rapid qualitative analysis as outlined in our methods. We have now removed the word "theme" to make this clearer.
9.A summary table of themes, subthemes, and patient quotations/examples is missing.	We have included a table of the key requirements identified in the analysis and

	exemplary quotes. We suggest that this table be a supplementary file (Appendix D).
10.Page 9: The discussion section is insufficiently structured. It should focus on discussing the differences in the previous symptom checklists used in the risk stratification system and the reasons for providing the information.	We have edited the discussion section and included additional content on the previous symptom checklist and the reasons for providing the information.<br><br>*It can be difficult to identify high-risk HNCs in England due to poor referral information.[19] This is important as HNCs can progress quickly – with even short delays in time to treatment being more likely to lead to reductions in survival than is the case for other common cancers such as breast and prostate [21]…*<br>*The HaNC-RC was developed for use by specialists to aid identification of high-risk patients [10]. Our data demonstrates that it can require a series of questions for clinicians to generate the responses needed for completion of HaNC-RC, and has highlighted the clarifications that may be needed to distinguish different symptoms.*
